# MRI-based radiomics approach for differentiation of hypovascular non-functional pancreatic neuroendocrine tumors and solid pseudopapillary neoplasms of the pancreas

**DOI:** 10.1186/s12880-021-00563-x

**Published:** 2021-02-23

**Authors:** Tao Song, Qian-Wen Zhang, Shao-Feng Duan, Yun Bian, Qiang Hao, Peng-Yi Xing, Tie-Gong Wang, Lu-Guang Chen, Chao Ma, Jian-Ping Lu

**Affiliations:** 1 Department of Radiology, Changhai Hospital, The Navy Medical University (Second Military Medical University), 168 Changhai Road, Shanghai, 200433 China; 2 GE Healthcare China, Pudong New Town, No.1 Huatuo Road, Shanghai, 210000 China

**Keywords:** Pancreatic neuroendocrine tumors, Solid pseudopapillary neoplasms of the pancreas, Radiomics, Magnetic resonance imaging

## Abstract

**Background:**

This study aims to investigate the value of radiomics parameters derived from contrast enhanced (CE) MRI in differentiation of hypovascular non-functional pancreatic neuroendocrine tumors (hypo-NF-pNETs) and solid pseudopapillary neoplasms of the pancreas (SPNs).

**Methods:**

Fifty-seven SPN patients and twenty-two hypo-NF-pNET patients were enrolled. Radiomics features were extracted from T1WI, arterial, portal and delayed phase of MR images. The enrolled patients were divided into training cohort and validation cohort with the 7:3 ratio. We built four radiomics signatures for the four phases respectively and ROC analysis were used to select the best phase to discriminate SPNs from hypo-NF-pNETs. The chosen radiomics signature and clinical independent risk factors were integrated to construct a clinic-radiomics nomogram.

**Results:**

SPNs occurred in younger age groups than hypo-NF-pNETs (*P* < 0.0001) and showed a clear preponderance in females (*P* = 0.0185). Age was a significant independent factor for the differentiation of SPNs and hypo-NF-pNETs revealed by logistic regression analysis. With AUC values above 0.900 in both training and validation cohort (0.978 [95% CI, 0.942–1.000] in the training set, 0.907 [95% CI, 0.765–1.000] in the validation set), the radiomics signature of the arterial phase was picked to build a clinic-radiomics nomogram. The nomogram, composed by age and radiomics signature of the arterial phase, showed sufficient performance for discriminating SPNs and hypo-NF-pNETs with AUC values of 0.965 (95% CI, 0.923–1.000) and 0.920 (95% CI, 0.796–1.000) in the training and validation cohorts, respectively. Delong Test did not demonstrate statistical significance between the AUC of the clinic-radiomics nomogram and radiomics signature of arterial phase.

**Conclusion:**

CE-MRI-based radiomics approach demonstrated great potential in the differentiation of hypo-NF-pNETs and SPNs.

## Background

Solid pseudopapillary neoplasms (SPNs) of the pancreas are rare pancreatic tumors, accounting for about 2%-3% of pancreatic neoplasms [1, 2]. SPNs are frequently seen in female patients and are typically manifested as large well-bordered round or round-like masses mostly with a clear capsule. Cystic solid changes are often observed as well as bleeding and calcification [2, 3]. Pancreatic neuroendocrine tumors (pNETs) also belong to rare pancreatic tumors [4, 5]. PNETs account for about 1%-5% of all pancreatic neoplasms [4]. They are classified as functional pNETs and nonfunctional pNETs (NF-pNETs) according to the appearance of hormone secretion-related syndrome [6]. PNETs usually appear as solid well-circumscribed avidly enhancing mass [7]. However, atypical appearances of pNETs have been described such as hypovascular lesions and solid-cystic components, which may mimic SPNs [7]. The prognosis of pNETs differs from that of SPNs significantly. Though both have malignant potential, SPNs are more indolent than pNETs and are considered to have better survival outcomes than pNETs. The overall survival of SPNs is approximately 95% at 5 years [8] while that of pNETs is 65% at 5 years after complete surgical resection [9, 10]. Moreover, the treatment strategies are different for those two tumors. Surgery is the only curative treatment for SPNs and is also the standard treatment for localized pNETs [11–14]. But observation may be considered for small and low-grade NF-pNETs [15]. The treatment for metastatic pNETs is multimodal, including primary resection, target therapies such as everolimus, peptide radioreceptor therapy, and systemic chemotherapy [16]. Whereas, these systemic treatment strategies are not applied to SPNs.

It is crucial to differentiate pNETs from SPNs preoperatively so that the possible treatment and follow-up care could be planned. However, as the incidence of these two tumors are low, there is a paucity of literature deliberating the distinction between SPNs and pNETs [17–19], much less hypovascular NF-pNETs (hypo-NF-pNETs). Magnetic resonance imaging (MRI) is a valuable imaging approach for patients suspected of pancreatic neoplasms. MRI can present more soft-tissue characteristics than computed tomography (CT), and has the potential to assess functional and metabolic features of tumors. Radiomics is a novel image postprocessing technology which allows the high-throughput extraction of quantitative imaging features from radiologic images, providing detailed descriptions of tumor characteristics and crucial insights into tumor heterogeneity [20–22]. It has shown promising value in regard of tumor differentiation [23, 24]. Consequently, MRI-based radiomics approach may allow more effective features of neoplasms. To date, research on radiomics differentiating SPNs and pNETs is still at the exploratory stage and few studies have been published. The purpose of our study was to investigate the value of radiomics parameters derived from contrast-enhanced MR (CE-MR) images in distinguishing SPNs from hypo-NF-pNETs. Moreover, we aimed to establish and validate an effective model and represent it with a nomogram to differentiate these two neoplasms.

## Methods

This study was a single-center retrospective study approved by the Committee on Ethics of Medicine of Changhai hospital and the requirement to obtain written informed consent was waived by the Committee on Ethics of Medicine of Changhai hospital. All methods were performed in accordance with the relevant guidelines and regulations.

### Patients

**Fig. 1:**
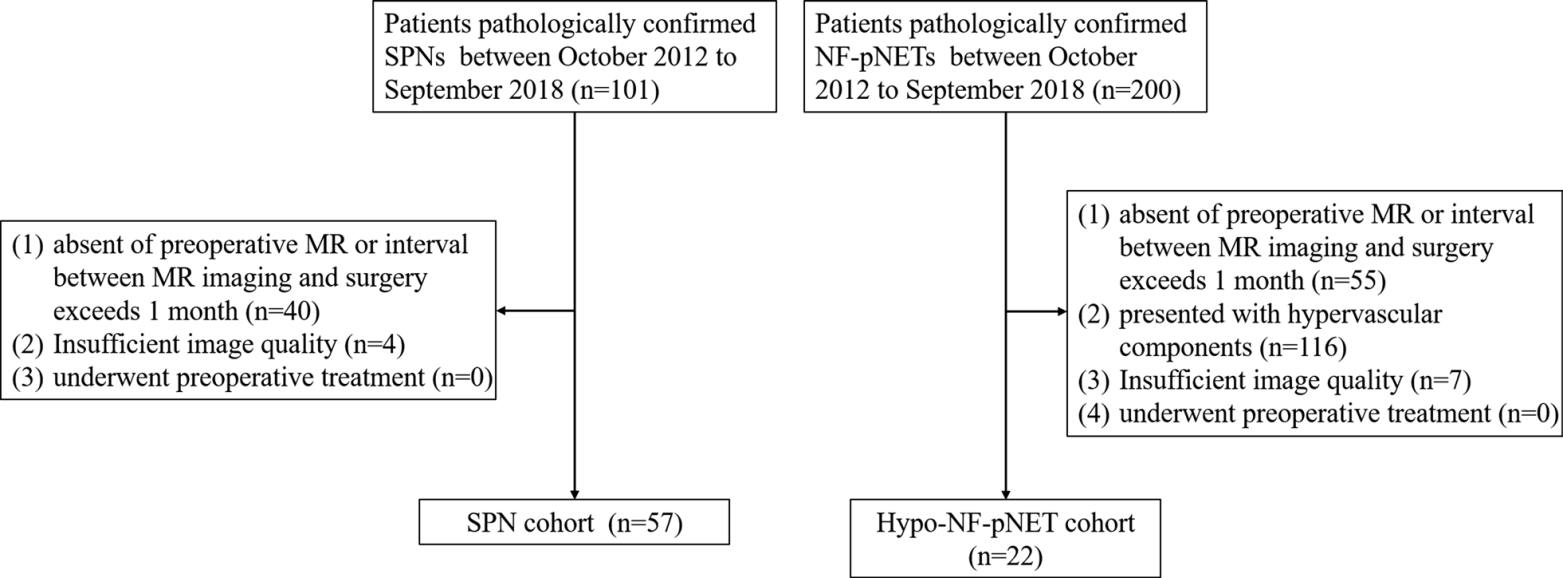
Flowchart of the patients’ recruitment

Patients pathologically proven as SPNs or NF-pNETs were identified through the computerized patient record system. The enrollment period was from October 2012 to September 2018. Those NF-pNET lesions which showed lower enhancement degrees than those of the adjacent pancreatic parenchyma at both arterial and portal phase were considered as hypo-NF-pNETs. The inclusion criteria was: patients underwent surgical excision and subsequent histopathologic examination. Exclusion criteria were as follows: (1) absent of preoperative MR; (2) the interval between MR imaging and surgery exceeds one month; (3) NF-pNETs presented with hypervascular pattern on contrast-enhanced MR images; (4) insufficient image quality for further assessment; (5) underwent preoperative treatment. Figure 1 represents the flowchart of the patients’ recruitment.

### MR imaging

MR imaging was performed by a 3.0 T MR system (Signa HDxt MR750 3.0 T, GE Healthcare, Milwaukee, WI; Skyra 3.0 T, Siemens, Erlangen, Germany) with a body coil coving the upper abdomen. The routine pancreatic MR protocol contained breath-hold single-shot fast-spin echo-coronal T2-weighted imaging (T2WI) (repetition time [TR]/echo time [TE] = 6316/87 ms; field of view [FOV] = 360 × 420 mm^2^; matrix = 224 × 270; flip angle = 90; slice thickness = 5 mm; slice gap = 1 mm), pre-contrast T1-weighted imaging (T1WI), three-phase contrast-enhanced fat-saturated T1WI (TR/TE = 2.58/1.18 ms; FOV = 440 × 440 mm^2^; matrix = 224 × 270; flip angle = 12; slice thickness = 5 mm; no slice gap) and diffusion-weighted imaging (DWI) (b-value = 0, 800 s/mm^2^). Gadolinium-diethylenetriamine pentaacetic acid (Gd-DTPA, Magnevist, Bayer Schering Pharma, Berlin, Germany) of a dose 0.2 mL/kg of was intravenous administrated for contrast media enhancement, with an injection rate of 2 mL/s. Contrast-enhanced images were acquired at 20-25 s (arterial phase), 60-70 s (portal phase) and 90-100 s (delayed phase) after contrast injection.

## Radiomic analysis

### Segmentation

We used artery phase to segment the region of interest (ROI), and the ROIs were transferred to precontrast T1WI, portal phase and delayed phase. One experienced radiologist (Reader1, S. T, with ten-years-experience in abdominal radiology) drawn the ROI on every slice of the tumors using ITK-SNAP software and got three-dimensional ROI. The ROI was delineated at the axial slice and carefully excluded the vessel and other tissues. In order to verify the consistency of segmentation, we randomly selected thirty images in the cohort and the same radiologist drawn the ROI again in the interval of two weeks. Meanwhile, another experienced radiologist (Reader 2, Z. QW, with six-years-experience in abdominal radiology) also segmented the tumor of the thirty images. Disagreements were resolved by consensus and were final confirmed by a specialist (L. JP with thirty-years-experience in abdominal radiology). Two times segmentation of Reader 1 was used to validate the intra-observer agreement of the radiomics feature. The first-time segmentation of Reader 1 and the segmentation of Reader 2 were used to test the inter-observer agreement of the radiomics features. The agreement test method was ICC analysis, and the feature with ICCs > 0.75 in both inter-and intra-observer test were remained.

### Feature extraction

The pyradiomics package was used to extract the radiomics features from precontrast T1WI, arterial phase, portal phase and delayed phase of postcontrast T1WI sequence. Before feature calculation, image preprocess was performed firstly, which mainly included three steps, voxel resampling, gray-level discretion, and image intensity normalization. We resampled the image to [1, 1, 1] voxel size to ensure the voxel was isotropic and the features were rotation invariant. Gray level discretion was helpful to reduce the computational consumption, and we discrete gray level with the bin width 20. Intensity normalization was conducted to enlarge the difference between the classes using $\mu \pm 3\sigma$ method [25]. Then three categories of features were obtained, which contained the first-order features, 2D shape features, texture features and high-order features.

### Feature selection and model construction

The subjects were divided into training cohort and validation cohort with the 7:3 ratio. The following description were all about the training cohort, unless emphasized validation cohort was used. Two feature selection methods were adopted to filter the features. We firstly used max-relevance and min-redundancy (mRMR) method to exclude most features and only kept thirty features which had least redundancy with each other feature and the most relevant with the targeted label [26]. Then the least absolute shrinkage and selection operator (LASSO) was performed to select the most predictive feature subset by choosing the optimized hyper-parameter $\uplambda$ which minimized the predictive bias. Then the remained features were used to construct the model using multivariate logistic regression, and the radiomics signatures were calculated for each subject. The radiomics signature was calculated by summing the features multiplying their corresponding coefficients. We built four model for precontrast T1WI, arterial phase, portal phase and delayed phase of postcontrast T1WI sequence respectively.

### Model validation

The receiver operating characteristic (ROC) analysis was used to validate the performance of the radiomics signatures based on four phases in both training cohort and validation cohort. In order to get more robust results, we performed least group out cross validation (LGOCV) with one hundred times to get the mean area under the curve (AUC) of the one hundred ROC curves, then used Mann–Whitney U test to compare the difference between different models. The model with best performance was picked to build the clinic-radiomics nomogram. Then we used decision curve to compare the clinical usefulness of model.

### Nomogram construction and validation

We examined group differences in terms of age, gender, CA19-9 level, tumor size, tumor location. Parameters with statistical significance were filtered using the univariate logistic regression, factors with *P* < 0.05 were kept and transferred into backward step-wise multivariable logistic regression analysis, Akaike information criterion as criterion to find the best model and independent clinical predictors. The final predictors and radiomics signature were integrated to construct the clinic-radiomics nomogram.

ROC analysis was performed to evaluate the performance of the nomogram. The calibration curves were plotted to calibrate the model, decision curve analysis was conducted to analyze the clinical utility of the nomogram.

### Statistical analysis

Statistical analyses were performed using C programming language. Continuous variables were showed as mean ± standard deviation (SD) while categorical variables presented as numbers or percentages. Variance homogeneity and normal distribution were checked for continuous variables. Differences between groups were assessed using independent samples t test for continuous variables if they are conformed to normal distribution and the Wilcoxon rank sum test were performed for the remaining continuous variables. The categorical features were compared by the chi-squared test or the Fisher exact test. Logistic regression analysis was used to examine associations and find independent clinical predictors of the tumor. The ROC curve was performed to assess the diagnostic value of the radiomics signatures and the nomogram in both training and validation cohorts, providing the AUC values, 95% confidence interval (CI), sensitivity, specificity, and cut-off values. The calibration curves and Hosmer–Lemeshow test were used to calibrate the model, whereas decision curve analysis (DCA) was conducted to analyze the clinical utility of the nomogram. DeLong’s test was conducted to compare which model had the best performance. Two-tailed *P* values were always computed and a *P *value of less than 0.05 was considered to be statistically significant.

## Results

### Clinical characteristics

Fifty-seven patients with SPNs and twenty-two patients with hypo-NF-pNETs were included in this study. The baseline characteristics of these enrolled patients were summarized in Table 1. SPNs occurred in younger age groups than hypo-NF-pNETs and showed a clear preponderance in females. The mean age was 34.2 ± 11.8 years in patients with SPNs and was 47.2 ± 12.8 years in patients with hypo-NF-pNETs (*P* < 0.0001). There were thirteen (22.8%) males and forty-four (77.2%) females in the SPNs group and eleven (50%) males and eleven (50%) females in the hypo-NF-pNETs group (*P* = 0.0185). No significant differences were found with respect to the CA199 levels, tumor size, or tumor location and these factors were not involved into the final clinic-radiomics model.

**Table 1:** Baseline Characteristics of Patients with SPNs and hypo-NF-PETs

Variable	SPNs (n = 57)	hypo NF-pNENs (n = 22)	*P* value
Age (years), mean [range]	34.2 ± 11.8	47.2 ± 12.8	< 0.0001*
Gender			0.0185*
Male	13	11	
Female	44	11	
CA19-9 (μg/L, median, range)	8.84 (2.00, 185.65)	7.82 (2.00, 48.95)	0.4883
Tumor size (mm)	45.8 ± 31.3	37.5 ± 24.8	0.4278
Tumor location			0.4419
Head or neck	20	11	
Body	11	4	
Tail	26	7	
Grade of differentiation, n (%)			
G1	–	6	
G2	–	11	
G3	–	5	

SPN, solid pseudopapillary neoplasm; Hypo-NF-pNET, hypovascular pancreatic neuroendocrine tumor

^*^Statistically significant difference; *p* < 0.05 was considered as the threshold for statistical significance for all tests

### Building and evaluation of radiomic signatures of four phases

**Fig. 2:**
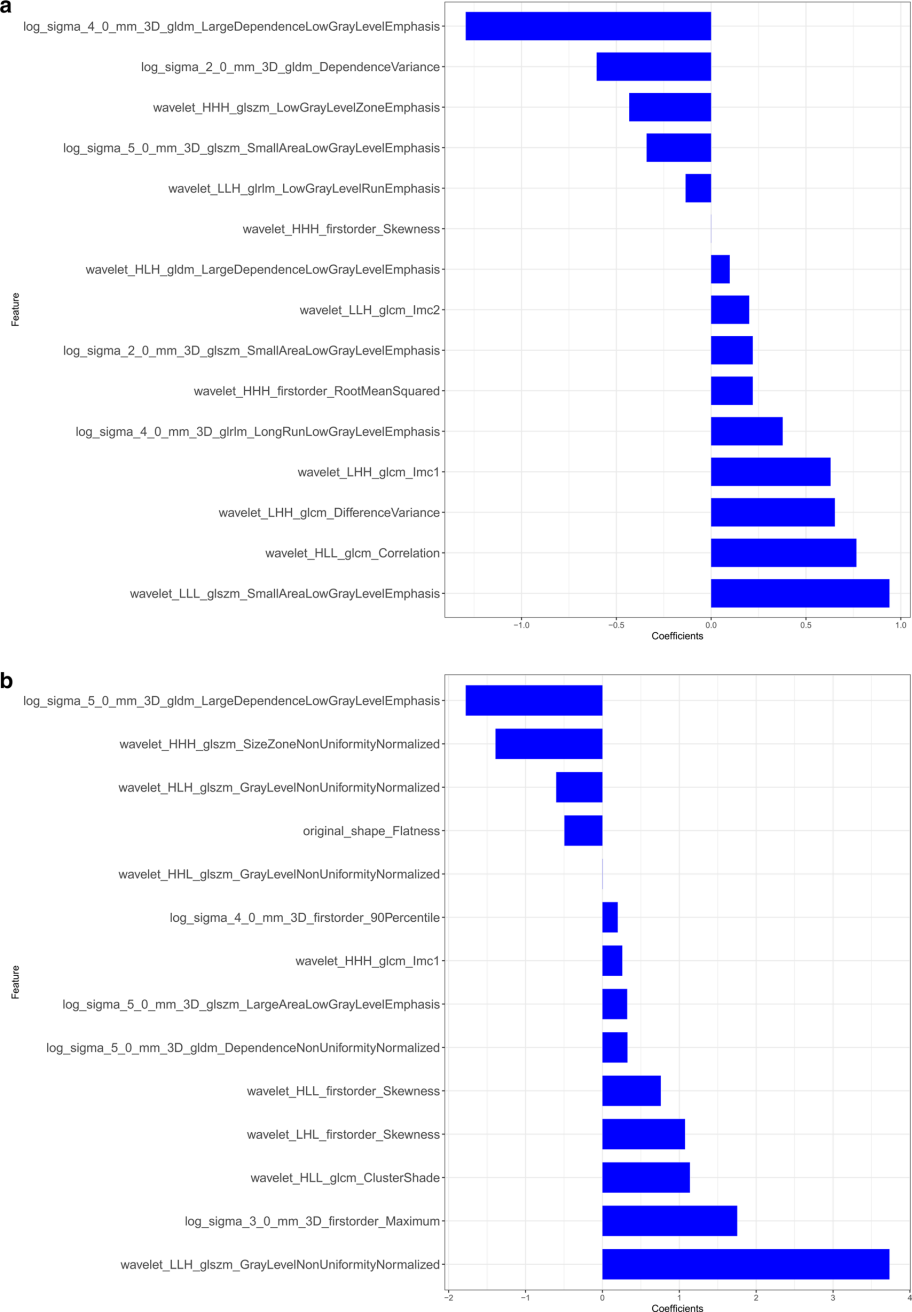

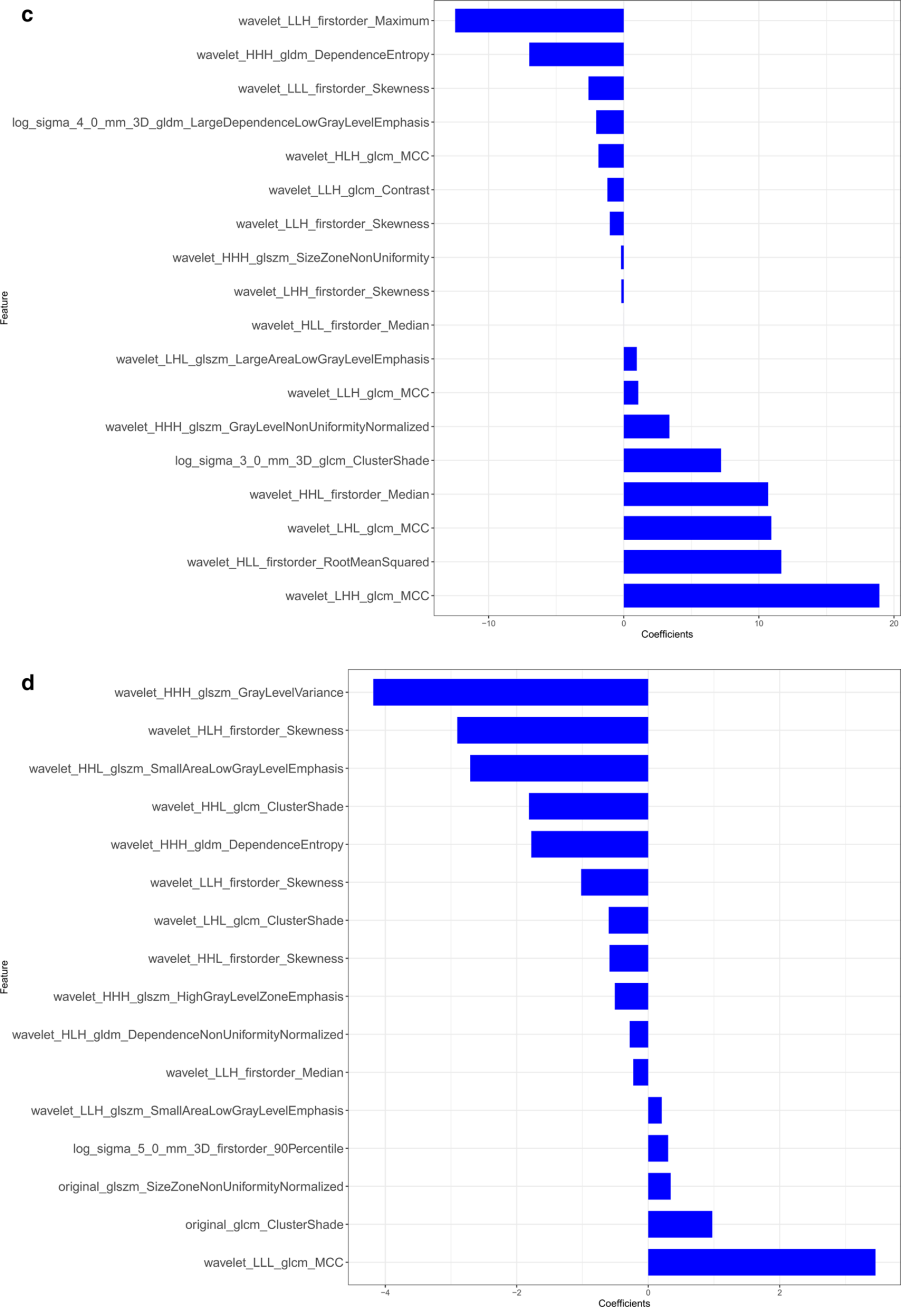
The selected radiomics features from LASSO regression and their associated coefficients. **a** Precontrast T1WI, **b** arterial phase, **c** portal phase, **d** delayed phase

**Fig. 3:**
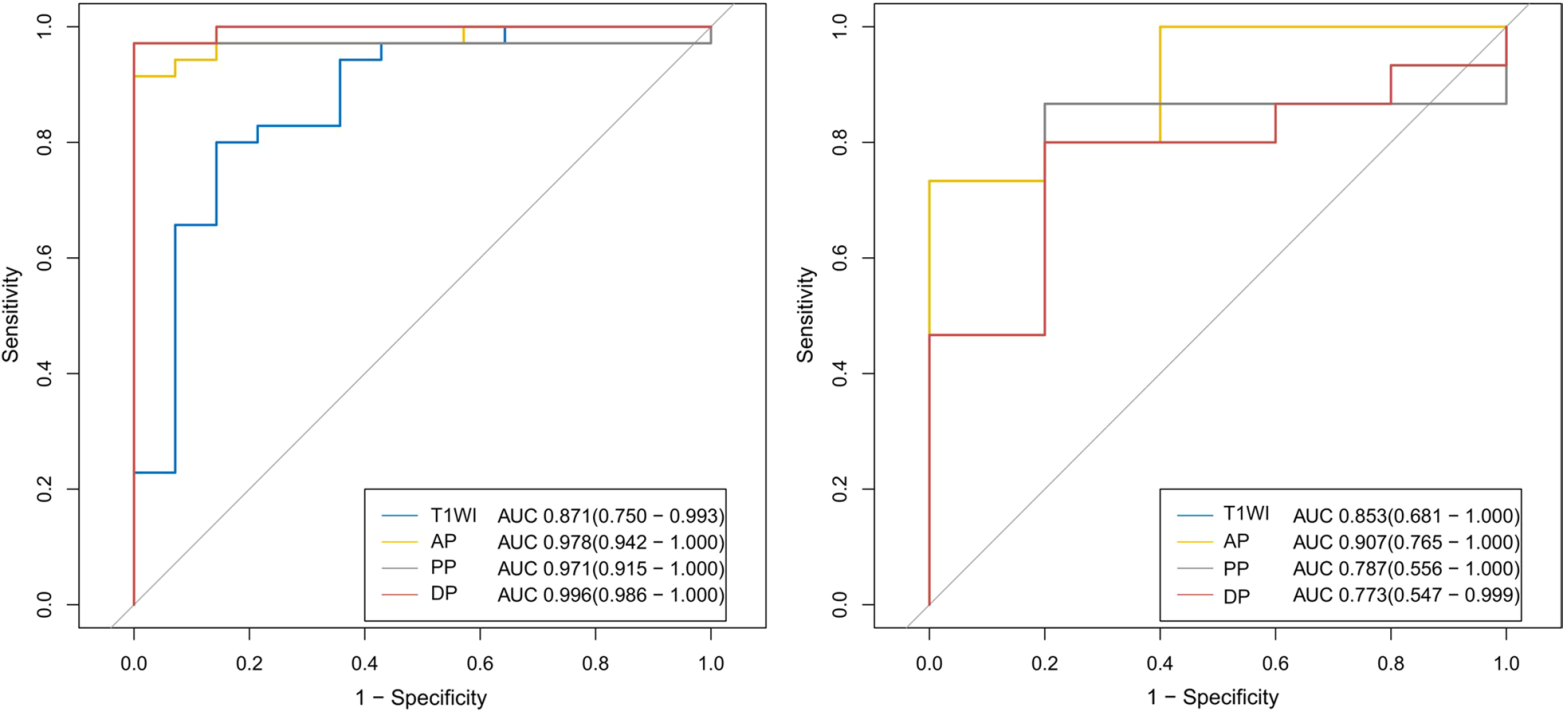
ROC curve of radiomics signatures extracted from CE-MRI for the training and validation cohort. AP, arterial phase; PP, portal phase; DP, delayed phase

We extracted 396 radiomics features from the ROIs of T1WI, arterial phase, portal phase and delayed phase of post-contrast T1WI sequences, including 42 first-order features, 9 shape features and 345 texture features. We used mRMR method and LASSO regularization to exclude features and determine the most predictive subset features. Fifteen features were selected from the T1WI sequence, fourteen features were selected from the arterial phase, eighteen features were selected from the portal phase and sixteen features were selected from the delayed phase. The selected features and their associated coefficients were displayed in Fig. 2. The corresponding radiomics signatures of the four phases were built using the radiomics features selected. The AUC values of radiomics signatures for the training and validation cohort were shown in Fig. 3. The radiomics signatures showed favorable discriminatory abilities in the training set with AUC values over 0.850 (precontrast T1WI 0.871 [95% CI 0.750–0.993], arterial phase 0.978 [95% CI 0.942–1.000], portal phase 0.971 [95% CI 0.915–1.000]), delayed phase 0.996 [95% CI 0.986–1.000]). In the validation cohort, the AUC values were 0.853 (95% CI 0.681–1.000) for precontrast T1WI, 0.907 (95% CI 0.765–1.000) for arterial phase, 0.787 (95% CI 0.556–1.000) for portal phase and 0.773 (95% CI 0.547–0.999) for delayed phase respectively.

### Establish and validation of the clinic-radiomics nomogram

**Fig. 4:**
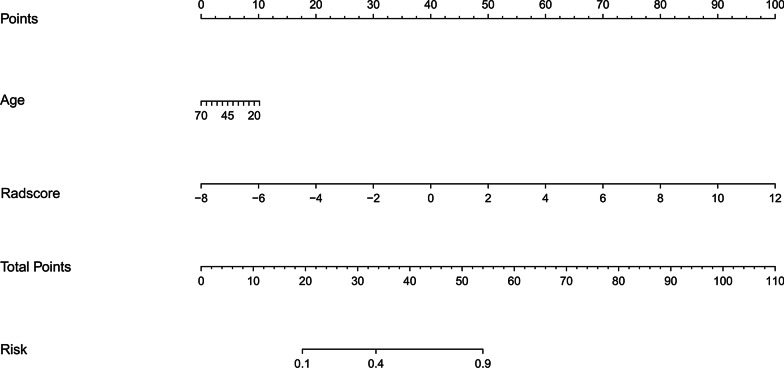
Quantitative nomogram for the discrimination of SPNs and hypo-NF-pNETs. The closer the risk is to 0.9, the more likely the tumor is to be SPN, and the closer the risk is to 0.1, the more likely it is to be hypo-NF-pNETs. Radscore was calculated by summing the selected features weighted by their coefficients. The final formula of radscore is: Radscore = -0.601 × wavelet_HLH_glszm_GrayLevelNonUniformityNormalized + 0.326 × log_sigma_5_0_mm_3D_gldm_DependenceNonUniformityNormalized + -1.391 × wavelet_HHH_glszm_SizeZoneNonUniformityNormalized + 1.138 × wavelet_HLL_glcm_ClusterShade + -1.778 × log_sigma_5_0_mm_3D_gldm_LargeDependenceLowGrayLevelEmphasis + 1.074 × wavelet_LHL_firstorder_Skewness + 0.199 × log_sigma_4_0_mm_3D_firstorder_90Percentile + 0.002 × wavelet_HHL_glszm_GrayLevelNonUniformityNormalized + 3.734 × wavelet_LLH_glszm_GrayLevelNonUniformityNormalized + 0.76 × wavelet_HLL_firstorder_Skewness + 0.259 × wavelet_HHH_glcm_Imc1 + 0.322 × log_sigma_5_0_mm_3D_glszm_LargeAreaLowGrayLevelEmphasis + 1.754 × log_sigma_3_0_mm_3D_firstorder_Maximum + -0.495*original_shape_Flatness + 2.127

**Fig. 5:**
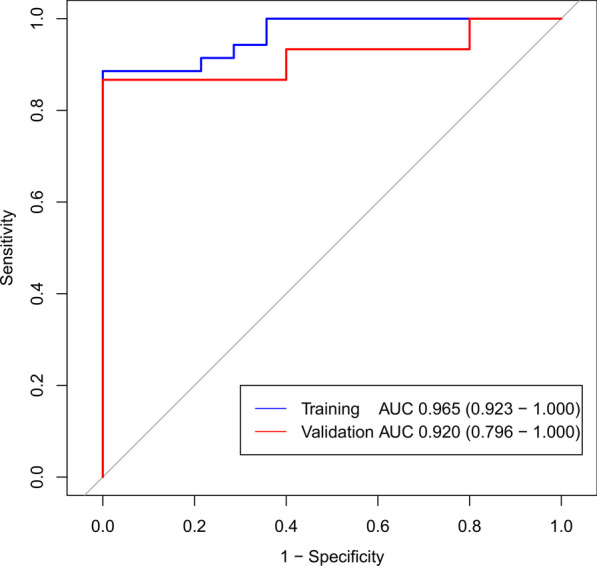
ROC curve of the established nomogram

**Fig. 6:**
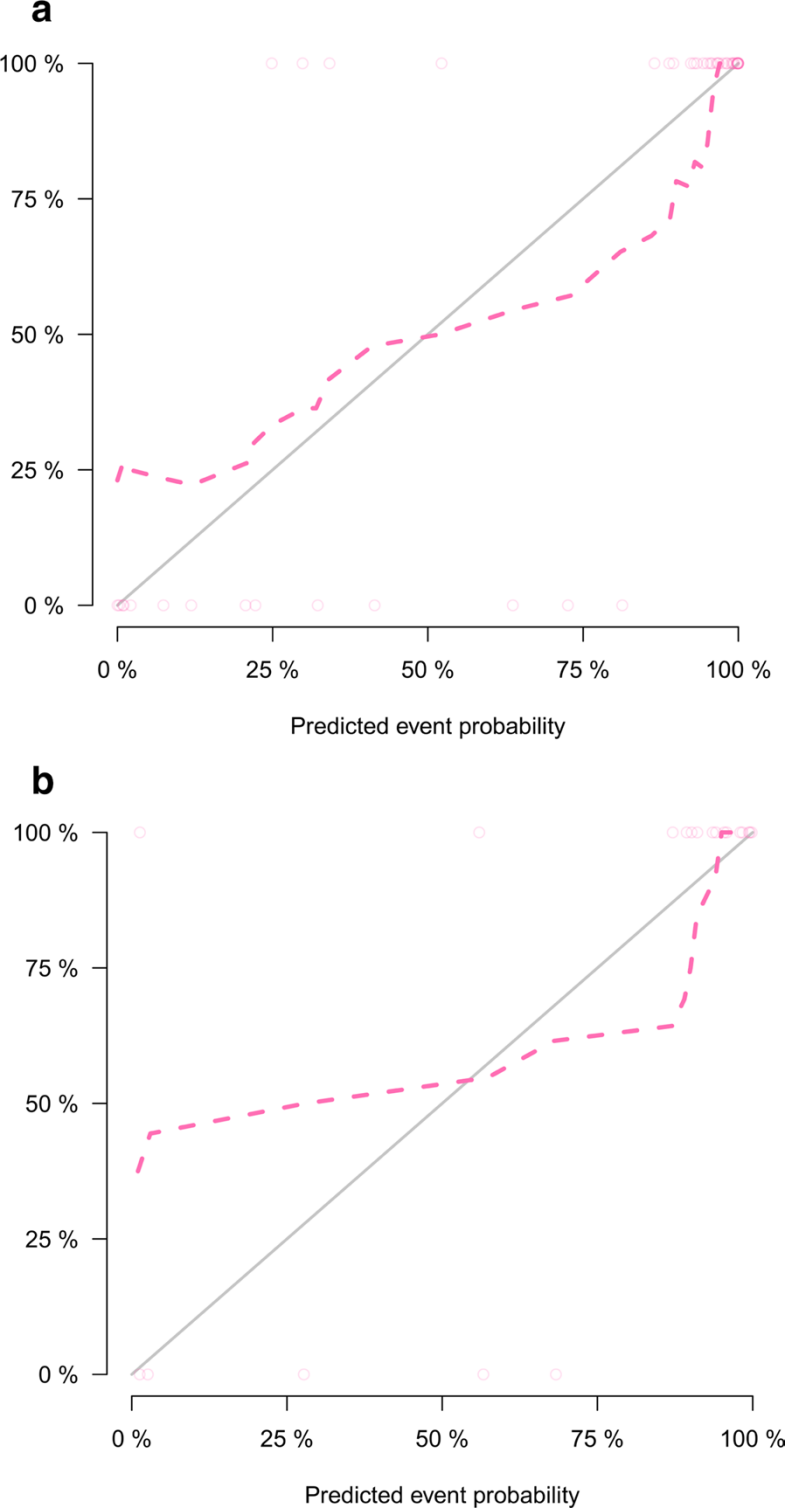
Calibration curves of the nomogram. **a** Training cohort, **b** validation cohort

**Fig. 7:**
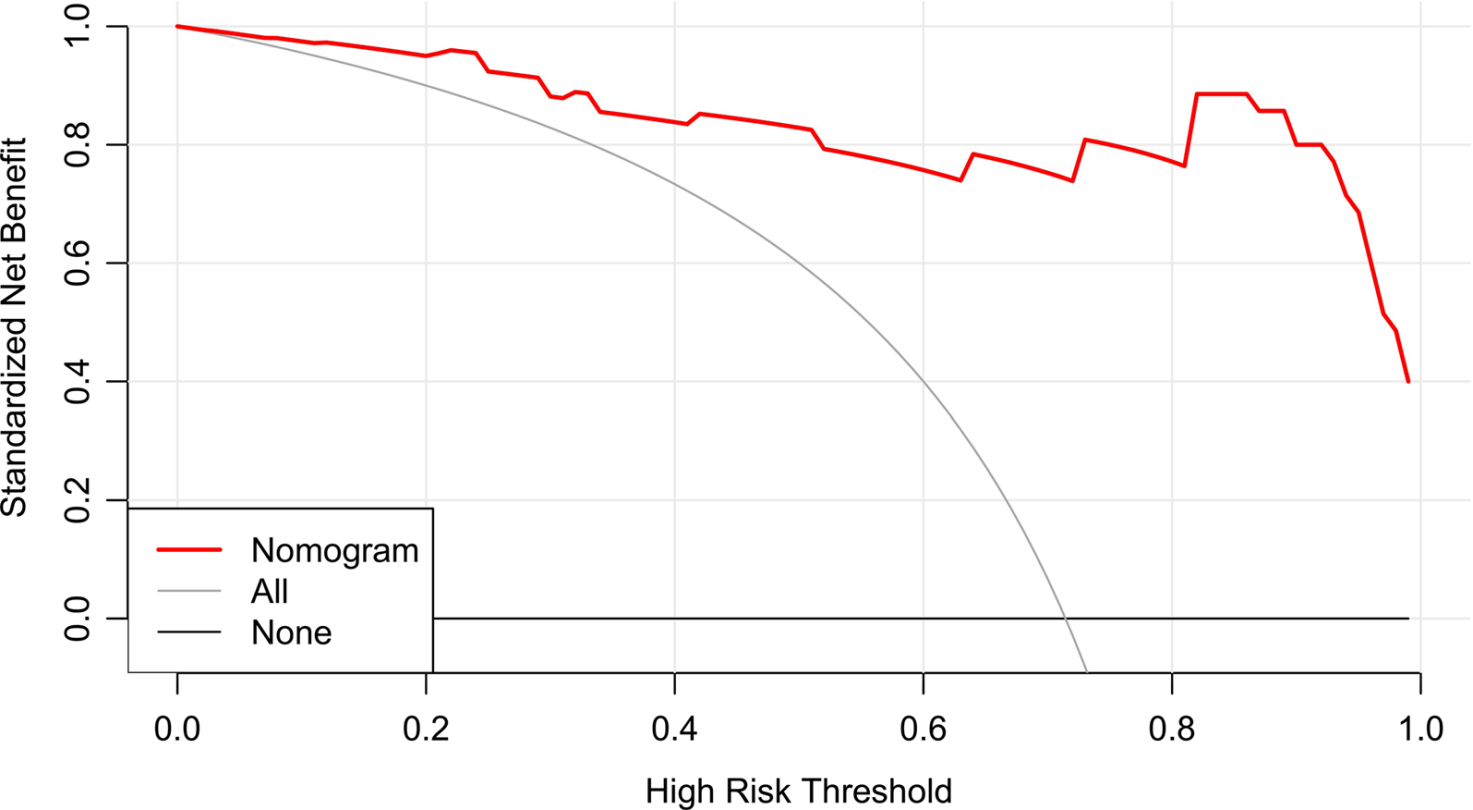
The decision curve analysis for the nomogram. The gray and black line represent the treat-all and treat-none scheme respectively

With AUC values above 0.900 in both training and validation cohort, the radiomics signature of the arterial phase was picked to build the nomogram. Accordingly, radscore was calculated by summing the selected features weighted by their coefficients. Logistic regression analysis revealed that age was a significant independent factor for the differentiation of SPN and hypo-NF-pNETs (*P* = 0.005). Consequently, a quantitative nomogram involving these two variables was established (Fig. 4). The nomogram showed sufficient performance for discriminating SPNs and hypo-NF-pNETs with AUC values of 0.965 (95% CI 0.923–1.000) and 0.920 (95% CI 0.796–1.000) in the training and validation cohorts, respectively (Fig. 5). The calibration curves of the nomogram were displayed in Fig. 6. The Hosmer–Lemeshow test indicated that the nomogram was acceptable in training cohorts (*P* = 0.33) but showed statistical significance in validation cohorts (*P* = 0.005). The nomogram yielded an accuracy of 91.8% (sensitivity, 100.0%; specificity, 77.8%) in the training cohort and 90.0% (sensitivity, 100.0%; specificity, 71.4%) in the validation cohort. As for the clinical utility, the decision curve analysis for the nomogram displayed that the nomogram would offer a certain higher overall benefit than both treat-all and treat-none scheme (Fig. 7). We further performed Delong Test to compare the diagnostic performance of the clinic-radiomics nomogram and radiomics signature of arterial phase. These two models showed roughly similar ability in differentiation of SPNs and hypo-NF-pNETs (*P* = 0.52 in training cohort and *P* = 0.47 in validation cohort).

## Discussion

With no representative clinical symptoms, the identification of NF-pNETs is sometimes harder than functional pNETs. NF-pNETs may present with variable imaging characteristics such as hypo-/iso-arterial phase enhancement and cystic degeneration, making it difficult to differentiate pNETs from SPNs [27–29]. As the therapeutic strategies and prognosis differ between them, the correct differentiation of these two tumors is of vital importance in clinical practice. In present study, we explored the feasibility of MR-based radiomics analysis for the differentiation of hypo-NF-pNETs and SPNs and constructed a clinic-radiomics nomogram to improve the diagnostic accuracy. Both the radiomics signature of arterial phase and the clinic-radiomics nomogram showed good accuracy in tumor differentiation and offered sufficient clinical utility. To the best of our knowledge, this is the first attempt to distinguish SPNs from hypo-NF-pNETs by means of MR-based radiomics approach.

In regard to data of our study, patients’ age and gender differed in SPNs and hypo-NF-pNETs. SPNs predominantly occurred in young women with mean age of 34.2 years. Meanwhile, age was an independent predictor comprising the nomogram as well as radiomics signatures of arterial phase. These data were consistent with the previous findings [17]. However, the age in univariate logistic regression had OR > 1, which means the older patient had higher risk score to be hypo-NF-pNETs. But in multivariate logistic regression, OR < 1 (OR 0.92 [0.87, 0.98], *P* = 0.005). In our knowledge, we thought maybe age was affected by other variables. This need to be further explored in future study.

Wang et al. [18] reported that SPNs usually present with younger age, a women preference and CT features involving an oval shape, “floating cloud” sign, calcification, and lower frequencies of metastases compared with hypovascular pNETs. The AUC value of the combined features (lower age, “floating cloud” sign, and calcification) is 0.865 for differentiating SPNs from hypovascular pNETs. However, it’s worth mentioning that traditional imaging features are mostly analyzed on the aspect of quality but not quantity, and the features are inevitably subjective, which may to some extent have an influence on the objectiveness of the findings. Thus, in our study, we chose the radiomics approach, which was recognized to provide robust imaging biomarkers of precision medicine. The AUC value of our radiomics nomogram, with 0.965 in training cohort and 0.920 in validation cohort, was higher than that of the model built by Wang [18] which was based on traditional imaging features. Our results suggested that radiomics approach might enrich image interpretations and improve the diagnostic accuracy.

MR is widely applied in clinical practice and played increasingly crucial role in stimulation of precise medicine with the advantage of radiation-free, easily-obtained, multi-sequencing and high soft tissue resolution. Li et al. [19] indicated that texture analysis could sensitively distinguish between NF-pNETs and SPNs on MRI, and parameters extracted from DCE-T1WI + fs images were informative for differentiation of neoplasms. Our data were consistent with their findings. But the enrolled patients of their research included both hypervascular and hypo-NF-pNETs. Considering it is typically not hard to distinguish hypervascular pNETs from SPNs in clinical practice, we believe the differentiation between hypo-NF-pNETs and SPNs would present more clinical importance. In our study, we excluded hypervascular NF-pNETs and focused on the discrimination of hypo-NF-pNETs and SPNs. We extracted not only texture features but also first-order and shape features by means of radiomic analysis. We also built the radiomics signatures of T1WI, arterial phase, portal phase and delayed phase of post-contrast T1WI respectively. In the training cohort, the four models all showed good discriminatory abilities with AUC values above 0.850 (T1WI 0.871, arterial phase 0.978, portal phase 0.971, delayed phase 0.996). According to the mean AUC of 100 ROC curves, the radiomics signatures of arterial phase and portal phase were significantly better than that of T1WI in training cohort, which emphasized the role of contrast enhancement in tumor differentiation. In the validation cohort, the radiomics signatures of T1WI and arterial phase were significantly better than that of delayed phase in validation cohort. As a result, the radiomics signature based on arterial phase was considered to be more robust than the other phases and was applied to establish the nomogram to distinguish SPNs and hypo-NF-pNETs. This finding suggested that the arterial phase might be a very helpful phase among CE-T1WI with radiomics features allows hypo-NF-pNETs to be distinguished from SPNs. The nomogram we built consisting of age and arterial radscore also confirmed the efficacy of MR-based radiomics features for differentiating NF-pNETs and SPNs.We believe that the radiomics approach has the potential to be applied in clinical practice after further modification with larger sample sizes.

We acknowledge that our study had several limitations. Firstly, this was a retrospective study at a single institution which may result in a selection bias. Secondly, the number of enrolled patients was relatively small for radiomic analysis, which was due to the low incidence of hypo-NF-pNETs and SPNs, as well as the strict inclusion and exclusive criteria to guarantee the robustness and accuracy of radiomics analysis. A multicenter program involving more patients would be warranted to further confirm the potential value of MR radiomics analysis in discriminating hypo-NF-pNETs and SPNs. Thirdly, the value of age in nomogram was limited, and the clinic-radiomics nomogram we built was not superior to simple arterial radiomics signature. Other clinical factors need to be further explored and the nomogram need to be modified in future prospective study.

## Conclusions

In conclusion, this study proposed a potentially reliable MRI-based radiomics approach for the differentiation of hypo-NF-pNETs and SPNs. It may be clinically accepted to improve the diagnostic accuracy and help in clinical decisions.

## Data Availability

The datasets used and/or analyzed during the current study are available from the corresponding author on reasonable request.

## Abbreviations

SPN: solid pseudopapillary neoplasm
pNET: pancreatic neuroendocrine tumor
NF-pNET: nonfunctional pancreatic neuroendocrine tumor
Hypo-NF-pNET: hypovascular nonfunctional pancreatic neuroendocrine tumor
MRI: magnetic resonance imaging
CT: computed tomography
CE-MRI: contrast-enhanced magnetic resonance imaging
T2WI: T2-weighted imaging
T1WI: T1-weighted imaging
FOV: field of view
DWI: diffusion-weighted imaging
ROI: region of interest
mRMR: max-relevance and min-redundancy
LASSO: least absolute shrinkage and selection operator
ROC: receiver operating characteristic
LGOCV: least group out cross validation
AUC: area under the curve
SD: standard deviation
CI: confidence interval
DCA: decision curve analysis

## Code availability

N/A.
